# Scattering Parameters Representing Imperfections in Precision Coaxial Air Lines

**DOI:** 10.6028/jres.094.015

**Published:** 1989

**Authors:** Donald R. Holt

**Affiliations:** National Institute of Standards and Technology, Boulder, CO 80303

**Keywords:** Bergman kernel, coaxial air line, conformal mapping, coupling, cubic splines, error sources, measurement contour, measurement precision, principal mode, scattering parameters, skin effect, surface roughness, telegraphist equations

## Abstract

Scattering parameter expressions are developed for the principal mode of a coaxial air line. The model allows for skin-effect loss and dimensional variations in the inner and outer conductors. Small deviations from conductor circular cross sections are conformally mapped by the Bergman kernel technique. Numerical results are illustrated for a 7 mm air line. An error analysis reveals that the accuracy of the scattering parameters is limited primarily by the conductor radii measurement precision.

## 1. Introduction

To accurately characterize imperfections of precision coaxial air lines, skin effect and surface roughness need to be considered. Skin effect is now well documented [1] and conductor surface finish has been studied in detail by Rice [2] and Ament [3] through the use of Fourier series methods. While Karbowiak [4] points out that Fourier analysis reveals useful knowledge of the spectral components which principally affect scattering parameters, it is also appropriate to examine local pointwise influence along the axial (z) coordinate. In this connection, Hill [5] developed perturbation expressions for the scattering parameters for a lossless circular air line. When the conductor surface exhibits transverse angular variation, Roimeliotes, Houssain and Fikioris report the effects of ellipticity and eccentricity on cutoff wave numbers [6].

The purpose of this paper is to develop numerically accurate pointwise coaxial air-line scattering parameters that account for skin effect loss and conductor surface variations in the transverse angular and axial directions. Following Schelkunoff [7], Reiter [8], Solymar [9] and Gallawa [10], generalized telegraphist equations for the principal mode are derived in section 2 for a circular air line. Transformation to forward and backward wave differential equations enables general solutions for the scattering parameters in section 3. To allow for conductor surface measurements along the z-axis, cubic spline polynomials provide a starting point for establishing pointwise recursion formulas of forward and backward waves in section 4. In section 5, the Bergman’s kernel technique is used to establish a conformal mapping for transforming noncircular conductors into equivalent circular conductors in correspondence to the principal mode. Computational results illustrating |*S*_11_| versus air-line length are given in section 6. An error analysis of the computational algorithms for the accuracy resolution of the measurement system is developed in section 7.

## 2. Generalized Telegraphist Equations for the Principal Mode

**Figure 1:**
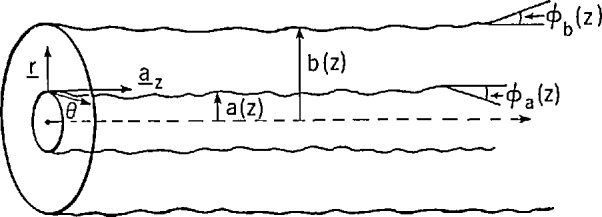
Coaxial air line.

Consider the coaxial air line in figure 1. With inner radius *a*(*z*) and outer radius *b*(*z*) the field components of primary interest are radial electric field (*E_r_*), angular magnetic field (*H*_θ_), and axial electric field (*E_z_*). We assume the fields *E_r_, H*_θ_, and *E_z_* are composed of TEM and TM modes, and coupling of the modes is caused by skin effect with variations of the conductor surfaces. From Appendix A boundary conditions for TM modes possess the form

$$E_{z}=-Z_{s}K\{\phi_{b}(z)\}H_{\theta }\;\text{at}\;r=b(z),$$ (1.0)

$$E_{z}=+Z_{s}K\{\phi_{a}(z)\}H_{\theta }\;\text{at}\;r=a(z),$$ (1.1)

where for instance,

$$K\{\phi_{b}(z)\}=\frac{1+\sin[\phi_{b}(z)]}{1+\tan[\phi_{b}(z)]}.$$ (1.2)

Appropriate Maxwell equations for determining transverse fields *E_r_* and *H_θ_* in the air dielectric region of the air line are [11]

$$\frac{\partial E_{r}}{\partial z}=-j\omega \mu \;H_{\theta }+\frac{\partial E_{z}}{\partial r}$$ (1.3)

$$\frac{\partial H_{\theta }}{\partial z}=-j\omega \epsilon \;E_{r}.$$ (1.4)

The parameters ω, *μ*, and *ϵ* are defined as radian frequency, permeability and permittivity, respectively, In addition the fields are assumed to vary with time according to the complex exponential function e*^jωt^*.

To find the generalized telegraphist equations it is convenient to assume the fields possess orthogonal expansions in *r* and *θ.* In view of TEM and TM modes together with impedance boundary conditions a set of orthogonal basis functions needs to be constructed from the Gram-Schmidt process. Assmning *E_r_, H_θ_*, and *E_z_* possess continuous first and second derivatives implies their expansions are absolutely and uniformly convergent [12]. In Appendix B these properties are used to rearrange the expansions into the form

$$E_{r}(r,\theta ,z)=\sum_{\left(n,p)=(0,0\right)}^{\left(\infty ,\infty \right)}V_{np}^{\left(1\right)}(z)e_{rnp}^{\left(1\right)}(r,\theta ,z)+V_{np}^{\left(2\right)}(z)e_{rnp}^{\left(2\right)}(r,\theta ,z)$$ (1.5)

$$H_{\theta }(r,\theta ,z)=\sum_{\left(n,p)=(0,0\right)}^{\left(\infty ,\infty \right)}I_{np}^{\left(1\right)}(z)h_{\theta np}^{\left(1\right)}(r,\theta ,z)+I_{np}^{\left(2\right)}(z)h_{\theta np}^{\left(2\right)}(r,\theta ,z)$$ (1.6)

where the superscripts (1) and (2) represent even and odd modes, respectively. We have

$$\left\{\begin{array}{c} e_{rnp}^{\left(1\right)}(r,\theta ,z)\\ e_{rnp}^{\left(2\right)}(r,\theta ,z) \end{array}\right\}=\frac{1}{N_{np}^{-1}(z)}f_{rnp}(r,z)\left\{\begin{array}{cc} \cos & k\theta \\ \sin & k\theta \end{array}\right\}.$$ (1.7)

In addition,

$$a_{\theta }h_{\theta np}^{\left(i\right)}(r,\theta ,z)=a_{z}\times a_{r}e_{rnp}^{\left(i\right)}(r,\theta ,z);i=1,2$$ (1.8)

and *N_np_*(*z*) denotes the norm of *f_rnp_*(*r,z*), that is,

$$N_{np}(z)=\int_{a(z)}^{b(z)}{\left\{f_{rnp}(r,z){\tilde{f}}_{rnp}(r,z)\right\}}^{1/2}dr,$$ (1.9)

where ^~^ stands for the complex conjugate. In particular for the TEM mode

$$e_{r00}^{\left(1\right)}(r,z)=\frac{1}{N_{00}(z)}\frac{1}{r},$$ (1.10)

$$N_{00}(z)={\left\{2\pi \ln\frac{b(z)}{a(z)}\right\}}^{1/2}.$$

Higher order modes are usual linear combinations of the first derivative Bessel functions ${J^{\prime }}_{n}$ and ${Y^{\prime }}_{n}$.

Following Reiter [8] by taking the inner product of eq (1.3) with the basis function ${\tilde{e}}_{rkq}^{\left(1\right)}$ yields

$$\int_{S(z)}\frac{\partial E_{r}}{\partial z}{\tilde{e}}_{rkq}^{\left(1\right)}dS=-j\omega \mu \int_{S(z)}a_{r}H_{\theta }\cdot \{a_{\theta }{\tilde{h}}_{\theta kq}^{\left(1\right)}\times a_{z}\}dS+\int_{S(z)}\frac{\partial E_{z}}{\partial r}{\tilde{e}}_{rkq}^{\left(1\right)}dS$$ (1.11)

where *S*(*z*) denotes the cross sectional air dielectric region between the conductors. The left side calls for differentiation of a variable surface integral and the second member of the right side integrates by parts.^1^ Hence, eq (1.11) evolves into the form

$$\begin{array}{l} \frac{d}{dz}\int_{S(z)}E_{r}{\tilde{e}}_{rkq}^{\left(1\right)}dS-\int_{S(z)}E_{r}\frac{\partial }{\partial_{Z}}{\tilde{e}}_{rkq}^{\left(1\right)}dS\\ =\oint {\;}_{L}\tan\{\theta_{b}(z)\}E_{r}{\tilde{e}}_{rkq}^{\left(1\right)}ds\\ -\oint {\;}_{L}\tan\{\phi_{a}(z)\}E_{r}{\tilde{e}}_{rkq}^{\left(1\right)}ds-j\omega \mu \int_{S(z)}H_{\theta }{\tilde{h}}_{\theta kq}^{\left(1\right)}dS\\ +\oint {\;}_{L}\{E_{z}[b(z),\theta ,z]-E_{z}[a(z),\theta ,z]\}{\tilde{e}}_{rkq}^{\left(1\right)}ds\\ -\int_{S(z)}E_{z}\frac{\partial }{\partial_{r}}{\tilde{e}}_{rkq}^{\left(1\right)}dS. \end{array}$$ (1.12)

To express *E_z_* in terms of the constructed basis function (*f_np_*), let the component of *E_z_* corresponding to *k* = 0 (*TM_op_* modes) be expressed as

$${\hat{E}}_{z0}=\pi \sum_{p=1}^{\infty }b_{0p}(z)\int_{r}f_{0p}(u)du.$$ (1.13)

From Maxwell’s equations the (0*p*) mode relation between *E_r_* and *E_z_* is

$${\hat{E}}_{r,o,p}(r,p)=-\frac{\gamma_{0p}}{k_{c,0p}^{2}}\frac{\partial {\hat{E}}_{z,0p}}{\partial r}$$ (1.14)

where γ_0*p*_ and *k*_*c*,0*p*_ are the propagation constant and cutoff frequency numbers, respectively. Using eqs (1.5), (1.13), and (1.14) yields

$$b_{0p}(z)=\frac{k_{c,0p}^{2}}{\gamma_{0p}}N_{np}^{-1}(z)V_{0p}^{\left(1\right)}(z).$$ (1.15)

Now substituting basis function definitions eq (1.7) and calling for the principal mode yields

$$\begin{array}{l} \frac{dV_{00}}{dz}-2\pi \sum_{p=0}^{\infty }\int_{a(z)}^{b(z)}\frac{f_{0p}(r,z)}{N_{0p}(z)}V_{0p}^{\left(1\right)}(z)\frac{\partial }{\partial_{Z}}\frac{{\tilde{f}}_{00}(r,z)}{{\tilde{N}}_{00}(z)}r\;dr\\ =-j\omega \mu \;I_{00}^{\left(1\right)}(z)-2\pi \;Z_{s}\;K[\phi_{b}(z)]\frac{{\tilde{f}}_{00}[b(z),z]}{{\tilde{N}}_{op}(z)}b(z). \end{array}$$

$$\sum_{p=0}^{\infty }I_{0p}^{\left(1\right)}(z)\frac{f_{0p}[b(z),z]}{N_{0p}(z)}-2\pi \;Z_{s}K[\phi_{a}(z)]\frac{{\tilde{f}}_{00}[a(z),z]}{{\tilde{N}}_{00}(z)}a(z).$$

$$\sum_{p=0}^{\infty }I_{0p}^{\left(1\right)}(z)\frac{f_{0p}[a(z),z]}{N_{0p}(z)}-\frac{2\pi }{{\tilde{N}}_{00}(z)}\sum_{p=1}^{\infty }\frac{k_{c,0p}^{2}}{\gamma_{0p}}\frac{V_{0p}^{\left(1\right)}(z)}{N_{0p}(z)}.$$

$$\begin{array}{l} \int_{a(z)}^{b(z)}\int_{r}f_{0p}(u)du\frac{\partial }{\partial r}{\tilde{f}}_{00}(r,z)r\;dr\\ +2\pi \tan[\phi_{b}(z)]\frac{{\tilde{f}}_{00}[b(z),z]}{{\tilde{N}}_{00}(z)}b(z)\sum_{p=0}^{\infty }\frac{f_{0p}[b(z),z]}{N_{0p}(z)}V_{0p}^{\left(1\right)}(z)\\ -2\pi \tan[\phi_{a}(z)]\frac{{\tilde{f}}_{00}[a(z),z]}{{\tilde{N}}_{00}(z)}a(z)\sum_{p=0}^{\infty }\frac{f_{0p}[a(z),z]}{N_{0p}(z)}V_{0p}^{\left(1\right)}(z). \end{array}$$ (1.16)

To derive the companion generalized telegraphist equation from eq (1.4), the procedure is almost identical. Taking the inner product of eq (1.4) with $h_{\theta kq}^{\left(1\right)}$ from eq (1.8) yields

$$\int_{S(z)}\frac{\partial H_{\theta }}{\partial z}h_{\theta kq}^{\left(1\right)}dS=-j\omega \in \int_{S(z)}a_{\theta }E_{r}\cdot a_{z}\times a_{r}{\tilde{e}}_{rkq}^{\left(1\right)}dS.$$ (1.17)

For the left side

$$\int_{S(z)}\frac{\partial H_{\theta }}{\partial z}{\tilde{h}}_{\theta kq}^{\left(1\right)}dS=\frac{d}{dZ}I_{kq}-\sum_{p=0}^{\infty }I_{kp}\int_{S(z)}\left\{a_{z}\times a_{r}{\tilde{e}}_{rkp}^{\left(1\right)}\right\}.$$

$$\frac{d}{dZ}\left\{a_{z}\times a_{r}{\tilde{e}}_{rkq}^{\left(1\right)}\right\}dS-\oint_{L}\tan\{\phi_{b}(z)\}H_{\theta }\;{\tilde{h}}_{\theta kg}^{\left(1\right)}dS+\oint_{L}\tan\{\phi_{a}(z)\}H_{\theta }\;{\tilde{h}}_{\theta kq}^{\left(1\right)}dS.$$ (1.18)

Since integration by parts obtains the relation

$$\int_{S(z)}e_{rkp}^{\left(1\right)}\frac{d}{dz}{\tilde{e}}_{rkp}^{\left(1\right)}dS=-\int_{S(z)}{\tilde{e}}_{rkp}^{\left(1\right)}\frac{d}{dz}e_{rkp}^{\left(1\right)}dS,$$ (1.19)

eq (1.17) takes on the form (setting *k*=*q*=0)

$$\begin{array}{l} \frac{dI_{00}}{dz}=-j\omega \epsilon V_{00}(z)-2\pi \sum_{p=0}^{\infty }I_{0p}(z)\int_{S(z)}{\tilde{e}}_{rkq}^{\left(1\right)}\frac{d}{dz}e_{r0p}^{\left(1\right)}dS\\ +b^{\prime }(z){\oint_{L}H_{\theta }\;\tilde{h}}_{\theta 00}^{\left(1\right)}dS-a^{\prime }(z){\oint_{L}H_{\theta }\tilde{h}}_{\theta 00}^{\left(1\right)}dS. \end{array}$$ (1.20)

Substituting eqs (1.6) and (1.10) into eq (1.20) gives

$$\begin{array}{l} \frac{dI_{00}^{\left(1\right)}}{dz}+2\pi \sum_{p=0}^{\infty }I_{0p}^{\left(1\right)}(z)\int_{a(z)}^{b(z)}\frac{{\tilde{f}}_{00}(r,z)}{{\tilde{N}}_{00}(z)}\frac{\partial }{\partial z}\frac{f_{0p}(r,z)}{N_{0p}(z)}r.\;dr\\ =-j\omega \epsilon \;V_{00}^{\left(1\right)}(z)\\ +2\pi \;b^{\prime }(z)\frac{{\tilde{f}}_{00}[b(z),z]}{{\tilde{N}}_{00}(z)}b(z)\sum_{p=0}^{\infty }I_{0p}^{\left(1\right)}(z)\frac{f_{0p}[b(z),z]}{N_{0p}(z)}\\ -2\pi \;a^{\prime }(z)\frac{{\tilde{f}}_{00}[a(z),z]}{{\tilde{N}}_{00}(z)}a(z)\sum_{p=0}^{\infty }I_{0p}^{\left(1\right)}(z)\frac{f_{0p}[a(z),z]}{N_{0p}(z)}. \end{array}$$ (1.21)

Examining eqs (1.16) and (1.21) reveals that continuous mode coupling occurs through the voltage and current transfer coefficients (left side), respectively, a phenomenon observed by Schelkunoff [7]. Skin effect coupling on the conductor surfaces was also reported by Schelkunoff and Gallawa [10]. When the air line is operated at frequencies appropriate to the principal mode, all TM modes attenuate rapidly below their cutoff frequencies. Consequently, dominant coupling occurs between the forward and backward waves of the principal mode.^2^ In this regard, eq (1.16) assumes the form

$$\begin{array}{l} \frac{dV_{00}}{dz}-2\pi \left\{\int_{a(z)}^{b(z)}\frac{1}{\left[2\pi \ln\frac{b(z)}{a(z)}\right]}1/2\frac{1}{r}\frac{\partial }{\partial z}\frac{1}{\left[2\pi \ln\frac{b(z)}{a(z)}\right]}1/2r\;dr\right\}V_{00}(z)=-j\omega \mu \;I_{00}(z)\\ -Z_{s}\left\{K[\phi_{b}(z)]\frac{1}{b(z)\ln\frac{b(z)}{a(z)}}+K[\phi_{a}(z)]\frac{1}{a(z)\ln\frac{b(z)}{a(z)}}\right\}I_{00}(z)\\ +\left\{\frac{b^{\prime }(z)}{b(z)\ln\frac{b(z)}{a(z)}}-\frac{a^{\prime }(z)}{b(z)\ln\frac{b(z)}{a(z)}}\right\}V_{00}(z), \end{array}$$ (1.22)

where the superscript, (1), has been dropped since only one mode is involved. Rearranging terms produces the expression

$$\frac{dV_{00}}{dz}+\{j\omega \mu +Z_{s}\kappa (z)\}I_{00}={T_{00}}^{\left(v\right)}(z)V_{00},$$ (1.23)

where

$$\kappa (z)=\frac{K[\phi_{b}(z)]}{b(z)}+\frac{K[\phi_{a}(z)]}{a(z)}\frac{1}{\ln\frac{b(z)}{a(z)}},$$ (1.24)

$$T_{00}^{\left(v\right)}(z)=\frac{1}{2}\left\{\frac{b^{\prime }(z)}{b(z)}-\frac{a^{\prime }(z)}{a(z)}\right\}\frac{1}{\ln\frac{b(n)}{a(z)}}.$$ (1.25)

The equation for current proceeds similarly. Equation (1.20) yields

$$\frac{dI_{00}}{dz}+\{j\omega \epsilon \;V_{00}+T_{00}^{\left(1\right)}(z)\;I_{00}$$ (1.26)

where the current transfer coefficient is defined as

$$T_{00}^{\left(l\right)}(z)=\frac{3}{2}\left\{\frac{b^{\prime }(z)}{b(z)\ln\frac{b(n)}{a(z)}}-\frac{a^{\prime }(z)}{a(z)\ln\frac{b(n)}{a(z)}}\right\}.$$ (1.27)

## 3. Conversion of Generalized Telegraphist Equations to Forward and Backward Wave Equations

Following Solymar [9] we define the amplitudes of the forward and backward waves $A_{00}^{+}$ and $A_{00}^{-}$ from the relations

$$\begin{array}{l} V_{00}={k_{0}}^{1/2}\{A_{00}^{+}+A_{00}^{-}\},\\ I_{00}={k_{0}}^{-1/2}\{A_{00}^{+}-A_{00}^{-}\}, \end{array}$$

where *k*_0_*=μ*/ϵ, the wave impedance. Substituting eq (2.1) into eqs (1.4) and (1.20) produces the expression

$$\frac{dA_{00}^{+}}{dz}+\left\{j\beta +\frac{Z_{s}\kappa (z)}{2k_{0}}\right\}A_{00}^{+}=T_{00}(z)A_{00}^{+}-\frac{Z_{s}\kappa (z)}{2k_{0}}A_{00}^{-}-\frac{1}{2}T_{00}(z)A_{00}^{-},$$ (2.2)

$$\frac{dA_{00}^{-}}{dz}-\left\{j\beta +\frac{Z_{s}\kappa (z)}{2k_{0}}\right\}A_{00}^{-}=T_{00}(z)A_{00}^{-}-\frac{Z_{s}\kappa (z)}{2k_{0}}A_{00}^{+}-\frac{1}{2}T_{00}(z)A_{00}^{+},$$ (2.3)

where *β*=*ω*√*μϵ*,

$$T_{00}(z)=\frac{1}{2}\{T_{00}^{\left(v\right)}(z)+T_{00}^{\left(1\right)}(z)\}.$$ (2.4)

In view of eqs (1.25) and (1.27) the last expression possesses the form

$$T_{00}(z)=\frac{1}{\ln\frac{b(n)}{a(z)}}\left\{\frac{b^{\prime }(z)}{b(z)}-\frac{a^{\prime }(z)}{a(z)}\right\}.$$ (2.5)

For a lossless airline, voltage and current transfer coefficients assume the form,

$$-T_{00}^{\left(v\right)}(z)=T_{00}^{\left(l\right)}(z)=\frac{1}{2}\frac{1}{\ln\frac{b(n)}{a(z)}}\left\{\frac{b^{\prime }(z)}{b(z)}-\frac{a^{\prime }(z)}{a(z)}\right\}.$$ (2.6)

Also, using Solymar’s assumption that reflection of the principal mode does not affect forward propagation of the principal mode yields expressions

$$\frac{dA_{00}^{+}}{dz}+j\beta A_{00}^{+}=0,$$ (2.7a)

and

$$\frac{dA_{00}^{+}}{dz}-j\beta A_{00}^{-}=\frac{1}{2}\frac{1}{\ln\frac{b(n)}{a(z)}}\left\{\frac{b^{\prime }(z)}{b(z)}-\frac{a^{\prime }(z)}{a(z)}\right\},$$ (2.7b)

which agree with Hill’s results [5].

Returning to eqs (2.2) and (2.3) and retaining Solymar’s assumption above leaves the terms $T_{00}(z)\;A_{00}^{\pm }$. Since coupling in this sense is meaningless, we drop the terms $T_{00}(z)\;A_{00}^{\pm }$ and obtain^3^

$$\frac{dA_{00}^{+}}{dz}+\left\{j\beta +\frac{Z_{s}\kappa (z)}{2k_{0}}\right\}A_{00}^{+}=0,$$ (2.8)

$$\frac{dA_{00}^{-}}{dz}-\left\{j\beta +\frac{Z_{s}\kappa (z)}{2k_{0}}\right\}A_{00}^{-}=\left\{\frac{Z_{s}\kappa (z)}{2k_{0}}+\frac{T_{00}(z)}{2}\right\}A_{00}^{+}.$$ (2.9)

To incorporate appropriate boundary conditions, let the incident wave be $A_{00}^{+}(0)=A_{0}$ with perfect termination at *z*=L, that is $A_{00}^{-}(L)=0$.

At this point the forward wave solution yields

$$A_{00}^{+}(z)=-A_{0}e^{-\{\int_{0}^{z}\{j\beta +\frac{Z_{s}\kappa (\zeta )}{2k_{0}}\}d\zeta \}},0⩽z⩽L$$ (2.10)

and, at z=0, the reflected wave expression

$$A_{00}^{-}(0)=-A_{0}\int_{0}^{L}\left\{\frac{Z_{s}\kappa (z)}{2k_{0}}+\frac{T_{00}(z)}{2}\right\}e^{-2\{\int_{0}^{z}\{j\beta +\frac{Z_{s}\kappa (\zeta )}{2k_{0}}\}d\zeta \}}dz$$ (2.11)

show general forms which remain to be useful for using conductor radii measurements.

From eqs. (2.6) and (2.7) the scattering parameters *S*_11_ are *S*_21_ are defined as follows:

$$S_{11}=\frac{A_{00}^{-}(0)}{A_{0}}\text{and}\;S_{21}=\frac{A_{00}^{+}(L)}{A_{0}}.$$ (2.12)

## 4. Cubic Spline Fitting of Conductor Radius Measurements

Underlying an accurate solution to $A_{00}^{+}$ and $A_{00}^{-}$ are two critical items: (a) fitting conductor radii measurements with acceptable error bounds and (b) expansion of all known functions in a systematic manner to sufficient powers of *z*.

To handle (a) consider cubic spline polynomials [13] for the inner (or outer) conductor measurements such that

$${\hat{C}}_{k-1}(z)={\hat{C}}_{0,k-1}+\cdots +{\hat{C}}_{3,k-1}z^{3}$$ (3.1)

where ${\hat{C}}_{k-1}(z)$ approximates *a*(*z*) or *b*(*z*), *z_k_*_−_*_1_*⩽*z*⩽*z_k_* such that *k* = 1,*…;N* and *Z_N_=L.* It is desirable to transform the cubic spline eq (3.1) over the interval [*z_k_*_−1_,*z_k_*] into the representation^4^

$$C_{k-1}(\zeta )=C_{0,k-1}+\cdots +C_{3,k-1}\zeta^{3},$$ (3.2a)

in such a way that the condition,

$$\frac{d^{2}C_{k-1}(\zeta )}{d\zeta^{2}}=\frac{d^{2}}{dz^{2}}{\hat{C}}_{k-1}(z),$$ (3.2b)

holds at *z=z_k−_*_1_ and *z=z_k_* where ζ*=z_k_*−z*_k_*_−_*_1_.* In addition we require

$$C_{k-1}(0)={\hat{C}}_{0,k-1},$$

and

$$C_{k-1}(z_{k}-z_{k-1})={\hat{C}}_{0,k-1}+\cdots +{\hat{C}}_{3,k-1}z_{k}^{3}$$ (3.2c)

such that ${\hat{C}}_{0,k-1}$ represents the measurement of *a*(*z*) or *b*(*z*) at z=z*_k_*_−1_.

To implement (b), recall that *E_r_*, *H_θ_*, and the second derivatives of *E_r_* and *H_θ_* are assumed to be analytic functions in *r*, *θ*, and *z*. Hence, the expansions of ${\left\{b(z)\ln\frac{b(z)}{a(z)}\right\}}^{-1}$ and ${\left\{a(z)\ln\frac{b(z)}{a(z)}\right\}}^{-1}$ can be rearranged in powers of *z*. In Appendix C the following expressions are derived over the interval [z*_m_*_−1_,z*_m_*]:

$$j\beta +\frac{Z_{s}\kappa (\zeta )}{-2k_{0}}≐\sum_{k=0}^{3}\delta_{k{,}_{m-1}}^{\left(1\right)}\zeta^{k},$$ (3.3)

and

$$\frac{Z_{s}\kappa (\zeta )}{2k_{0}}+\frac{T_{00}(\zeta )}{2}≐\sum_{k=0}^{3}\delta_{k,m-1}^{\left(2\right)}\zeta^{k},$$ (3.4)

where 0⩽ζ⩽*z*−*z_m_*_−1_.

To establish $A_{00}^{-}$ and $A_{00}^{+}$ at each point *z_n_* it is convenient to employ recursion relations. Inserting eq (3.3) into eq (2.6) and examining the interval *z*_1_⩽*z*⩽*z*_2_ yields

$$A_{00}^{+}(0;z)=A_{00}^{+}{(0;z}_{1}{)e}^{-\{\int_{0}^{z-z_{1}}\sum_{k=0}^{3}\delta_{k,1}^{\left(1\right)}\zeta^{\kappa }d\zeta \}}$$ (3.5)

where

$$A_{00}^{+}(0;z_{1})=A_{0}e^{-\{\int_{0}^{z_{1}}\sum_{k=0}^{3}\delta_{k,0}^{\left(1\right)}\zeta^{\kappa }d\zeta \}}$$ (3.6)

is the forward wave emerging at *z*=*z*_1_.

For the interval *z_N_*_−1_⩽*z*⩽*z* eq (3.5) immediately generalizes to the recursion relation

$$A_{00}^{+}(0;z)=A_{00}^{+}{(0;z}_{N-1}{)e}^{-\{\int_{0}^{z-z_{N-1}}\sum_{k=0}^{3}\delta_{k,N-1}^{\left(1\right)}\zeta^{\kappa }d\zeta \}}.$$ (3.7)

Proceeding to the backward wave $A_{00}^{-}$ by using eqs (3.3) and (3.4) in eq (2.7) for the interval *z*_1_⩽*z*⩽*z*_2_ produces the relation

$$A_{00}^{-}(0;z)=A_{00}^{-}(0;z_{1})-A_{00}^{+}(0;z_{1})\{e^{-j\beta_{z_{1}}\}\int_{0}^{z_{2}-z_{1}}\{\sum_{k=0}^{3}\delta_{k,1}^{\left(2\right)}\eta^{\kappa }\}}.e^{-2\{\int_{0}^{\eta }\{\sum_{k=0}^{3}\delta_{k,1}^{\left(1\right)}\zeta^{\kappa }d\zeta \}}d\eta$$ (3.8)

where the transformation *η =z—z*_1_ introduces the term e−*jβz*_1_. Now eq (3.8) also generalizes to the recursion relation

$$A_{00}^{+}(0;z_{N})=A_{00}^{-}(0;z_{N-1})-A_{00}^{+}(0;z_{N-1})e^{-j\beta z_{N-1}\int_{0}^{z_{N}-z_{N-1}}\{\sum_{k=0}^{3}\delta_{k,N-1}^{\left(2\right)}\eta^{k}\}}.\{e^{-2\{\sum_{k=0}^{3}\delta_{k,N-1}^{\left(1\right)}\frac{\eta^{k+1}}{k+1}\}}\}d\eta .$$ (3.9)

From Appendix C eq (3.9) assumes the solution

$$A_{00}^{-}(0;z_{N})≐A_{00}^{-}(0;z_{N-1})-A_{00}^{+}(0;z_{N-1})e^{-j\beta z_{N-1}}\cdot \sum_{k=0}^{8}v_{k,N-1}^{\left(2\right)}S_{k}\left\{-\left[2j\beta +\frac{Z_{s}}{k_{0}}C_{0,N-1}^{\left(2\right)}\right](z_{N}-z_{N-1)}\right\},$$ (3.10)

where

$$S_{k}[\alpha (z_{N}-z_{N-1})]=\int_{Z_{N-1}}^{Z_{N}}e^{\alpha Z}Z^{k}dk.$$ (3.11)

## 5. Conductor Surface Variations in the Transverse Coordinates

When the outer conductor is bored, circular cross sections are the exception rather than the rule. Most likely, an elliptical cross section evolves with some degree of rotation. Consequently, it is desirable to perform mechanical measurements of conductor radii in the transverse plane to characterize the deviation from circular cross sections. Since the principal mode is TEM in the transverse plane a direct conformal mapping of the measvurement contour into an equivalent circular contour eliminates any difficulty of solving Laplace’s equation for an irregular boundary. If an equivalent circular contour is found for each transverse measurement plane on the air line, a corresponding set of scattering parameters represents the original air line of measurement contours.

The solution of Laplace’s equation for a TEM mode with the inner conductor potential held to *V*_0_ and the outer conductor potential set at 0 is

$$\phi =V_{0}\frac{\ln\{r/b(z)\}}{\ln\{\alpha (z)/b(z)\}}.$$ (4.1)

We initially state that Riemann’s mapping theorem assures a mapping from the contour *L* to the unit circle and a particular expression for mapping evolves from the Bergman kernel expansion [14]. Thus, for a contour *L* centered at ζ_0_ = δe^iθ^=0 the Bergman kernel is defined as

$$B(0,\xi )=\sum_{v=0}^{\infty }P_{v}^{\sim }(0)P_{v}(\xi ).$$ (4.2)

The Szegö polynomials *P_v_*(ζ) are constructed to be orthogonal on the contour *L* and ζ is a complex variable in the region bounded by *L.* Computing the inner (or outer) conductor radius requires the expression [15]

$$c(z)=\frac{\ell_{0}(z)}{2\pi }\cdot \frac{1}{B(0,0)},\;c(z)=a(z)\;\text{or}\;b(z),$$ (4.3)

where *ℓ* defines the contour length of *L* at the point *z*. To see how eq (4.3) is constructed consider the differential line element on *L*,

$$ds^{2}=dx^{2}+dy^{2},$$

which in *ρ*, *θ* coordinates becomes

$$ds^{2}=\left\{\rho^{2}(\theta )+{\left[\frac{\partial \rho }{\partial \theta }\right]}^{2}\right\}d\theta^{2}.$$ (4.4)

Then the length of *L* is

$$\ell_{0}=\int_{0}^{2\pi }ds=\int_{0}^{2\pi }{\left\{\rho^{2}(\theta )+{\left[\frac{\partial \rho }{\partial \theta }\right]}^{2}\right\}}^{1/2}d\theta .$$ (4.5)

To find representations for the Szego polynomials, consider the following orthogonalization procedure. Let the matrix elements,

$$h_{pq}=\frac{1}{\ell_{0}}\int_{L}\zeta^{p}\xi^{q}ds=\frac{1}{\ell_{0}}\int_{0}^{2\pi }\rho^{p+q}(\theta ){\left\{\rho^{2}(\theta )+{\left[\frac{\partial \rho }{\partial \theta }\right]}^{2}\right\}}^{1/2}e^{i(p-q)\theta }d\theta ,$$ (4.6)

be defined for *p*⩾0 and *q*⩾0. Note ${\tilde{h}}_{pq}=h_{qp}$. Then following Kantorivich and Krylov [15] compute the determinant

$$D_{0}=1,D_{n}=\left|\begin{array}{cccc} h_{00} & h_{10} & \cdots & h_{n0}\\ \vdots & & & \vdots \\ h_{0n} & h_{1n} & \cdots & h_{nn} \end{array}\right|.$$ (4.7)

Hence, the Szegö polynomial is defined as

$$P_{n}(\zeta )=\frac{1}{{\left[D_{n-1}D_{n}\right]}^{1/2}}\left|\begin{array}{cc} h_{00} & h_{10}\cdots \cdots h_{n0}\\ h_{01_{:}} & h_{11}\cdots \cdots h_{n1}\\ h_{0,n-1} & h_{1,n-1}\cdots h_{n,n-1}^{:}\\ 1 & z\cdots z^{n} \end{array}\right|,$$ (4.8)

such that

$$\frac{1}{\ell_{0}}\oint_{L}P_{n}(\zeta ){\tilde{P}}_{m}(\zeta )d\zeta =\delta_{nm}.$$ (4.9a)

Carrying out the above procedures yields the conductor radii accurate to third order,

$$C≐\frac{\ell_{0}}{2\pi }\left\{1-\frac{{\tilde{h}}_{10}h_{10}}{{\tilde{D}}_{1}D_{1}}-\frac{\left({\tilde{h}}_{10}{\tilde{h}}_{21}-{\tilde{h}}_{11}{\tilde{h}}_{20})(h_{10}h_{21}-h_{11}h_{20}\right)}{D_{1}{\tilde{D}}_{2}{\tilde{D}}_{1}D_{2}}\right\},$$ (4.9b)

where

$$D_{1}=h_{11}-h_{01}h_{10},$$

and

$$D_{2}=(h_{11}h_{22}-h_{12}h_{21})-h_{10}(h_{01}h_{22}-h_{02}h_{21})+h_{20}(h_{01}h_{12}-h_{02}h_{11}).$$ (4.10)

A convenient property of the Szego coefficients for symmetric contours is found from eq (4.6). We have

$$h_{pq}=\frac{1}{\ell_{0}}\int_{0}^{\pi }\{1+{\left(-1\right)}^{p+q}(\theta )\{{\rho^{2}(\theta )+{\left[\frac{\partial \rho }{\partial \theta }\right]}^{2}\}}^{1/2}e^{i(p-q)\theta }d\theta .$$

For off diagonal elements

$$h_{pq}=0;p+q\;\text{odd},\;p\neq q.$$

Hence, any asymmetry in the contour *L* is expected to be noticeable through the off-diagonal elements *h_pq_.*

To find the equivalent circular conductor radius, integrals in eqs (4.5) and (4.6) need to be determined from measurements of *ρ* and *θ* on the contour *L.* Let the following cubic spline be defined

$${\hat{\rho }}_{k-1}(\theta )=\sum_{i=0}^{3}{\hat{\rho }}_{i,k-1}\theta^{i}$$ (4.11a)

such that ${\hat{\rho }}_{k-1}(\theta )$ approximates *a*(*θ,z*) or *b*(*θ,z*) over the interval *θ_k−_*_1_⩽*θ*⩽*θ_k_* for *k* = 1,*…, N*, i.e., *θ_N_* = 2*π*.

Following the same procedures as in the transformation from *z* to ζ in eqs (3.2a) to (3.2c) enables the cubic spline.

$$\rho_{k-1}(\phi )=\sum_{i=0}^{3}(\rho_{i,k-1})\phi^{i},$$ (4.11b)

to be constructed where Φ *=θ−θ_k−_*_1_ and *ρ*_0,_*_k_*_−1_ equals the measurement of *a*(*θ,z*) or *b*(*θ,z*) at *θ= θ_k_*_−1_.

Equations (4.5) and (4.11) yield an expression for length using the binomial expansion:

$$\ell_{0}=\int_{0}^{2\pi }\left\{\rho (\theta )+\frac{1}{2}\rho^{-2}(\theta ){\left[\frac{\partial \rho }{\partial \theta }\right]}^{2}-..\right\}d\theta .$$ (4.12)

Appendix D, taking into account the spline coefficients, produces

$$\ell_{0}≐\sum_{n=0}^{N-1}\int_{0}^{\theta_{n+1}-\theta_{n}}\left\{\sum_{k=0}^{3}P_{1,k,n}^{\left(1\right)}\theta^{k}+\frac{1}{2}\sum_{k=0}^{7}P_{2,k,n}^{\left(1\right)}\theta^{k}\right\}d\theta ,$$ (4.13)

$$\ell_{0}≐\sum_{n=0}^{N-1}\left\{\sum_{k=0}^{3}P_{1,k,n}^{\left(1\right)}\frac{{\left[\theta_{n+1}-\theta_{n}\right]}^{k+1}}{k+1}+\frac{1}{2}\sum_{k=0}^{7}P_{2,k,n}^{\left(1\right)}\frac{{\left[\theta_{n+1}-\theta_{n}\right]}^{k+1}}{k+1}\right\}.$$

Expressions for the coefficients *h_pq_* are developed also in Appendix D.

## 6. Computation Results

**Figure 2:**
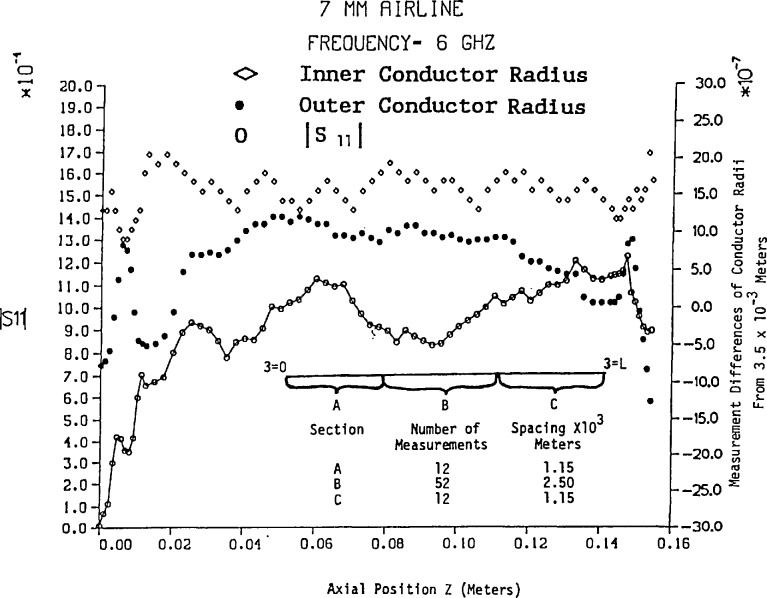
|*S*_11_| vs variable length of 7 mm lossless air line.

**Figure 3:**
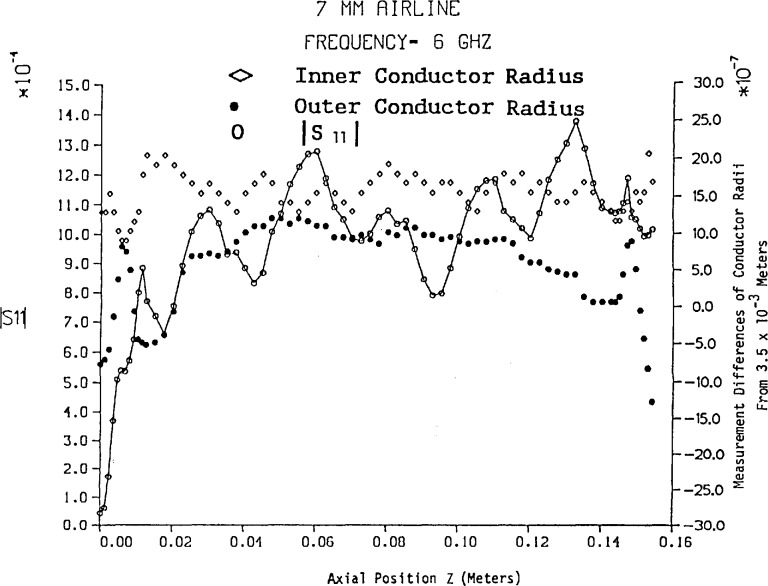
|*S*_11_| vs variable length of 7 mm air line with skin effect loss at 6 GHz.

**Figure 4:**
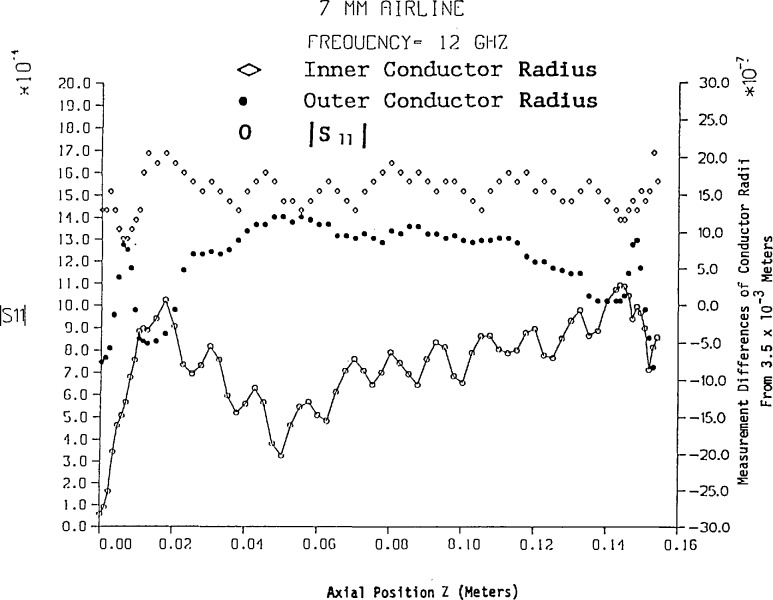
|*S*_11_| vs variable length of 7 mm air line with skin effect loss at 12 GHz.

**Figure 5:**
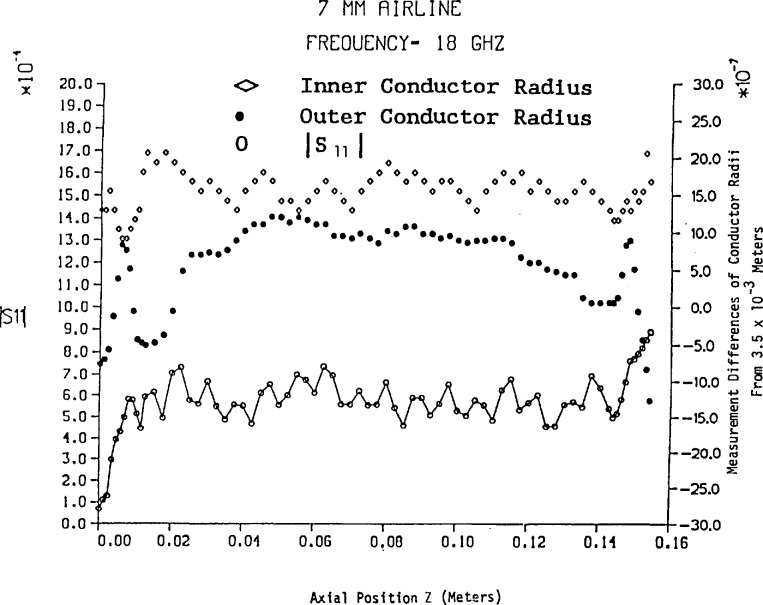
|*S*_11_| vs variable length of 7 nun air line with skin effect loss at 18 GHz

The amplitude of *S*_11_ has been computed from eqs (2.12) and (3.9) for a 7 mm air line approximately 15.6 cm in length using the frequencies 6, 12, and 18 GHz. In addition the number of conductor dimensional measurements in three sections of air line with variable spacing is shown in figure 2 and the results are illustrated in figures 2–5. Mean and standard deviation values of the conductor dimensional measurements are as follows:
Inner radiusOuter radiusMean (meters)0.1521×10^−2^0.3500×10^−2^Standard deviation (meters)0.2481×10^−6^0.6836×10^−^6

Figure 2 illustrates that conductor radius measurements near either end are more volatile—particularly the outer conductor. Figures 3, 4, and 5 reveal that changes in conductor radii in the *z*-axis provide the dominant contribution to |*S*_11_| while skin effect loss amplifies the in-phase and out-of-phase behavior of the lossless air line (as shown in fig. 2). In addition, skin effect loss affects the most significant digit of |*S*_11_| even for short lengths of line. On comparison of figures 3, 4, and 5 with Hill’s results [5], the most noticeable feature is the overall difference in magnitudes of *S*_11_, which evolves from a uniform inner conductor model and lossless boundary conditions in Hill’s work. Conformal mapping effects from elliptical measurement contours do not affect *S*_11_ and *S*_21_ unless the eccentricity is greater than 5×10^−6^ meters. However, if the inner conductor has an eccentric position with respect to the outer conductor, conformal mapping by Bergman’s kernel reveals scattering parameters *S*_11_ and *S*_21_ are noticeably affected.^5^

## 7. Error Analysis

### 7.1 Error Sources

Error sources that contribute to scattering parameters evolve from (a) spline interpolation with respect to *z*, (b) spline interpolation with respect to *θ*, (c) conformal mapping using the Bergman kernel, and (d) expressions for backward and forward waves.

To examine (a) and (b) consider the error bound from cubic spline interpolation theory [13],

$$|f(x)-S\Delta_{k}(x)|⩽\frac{5}{2}[\Delta_{k}^{2}\;\mu (f^{\prime \prime }(x);|\Delta_{k}|)],$$ (6.1)

where *S*Δ*_k_*(*x*) defines a cubic spline, *μ*, signifies the modulus of continuity,^6^ *f*″ denotes the second derivative of the function *f*, and Δ*_k_* stands for the mesh size between arguments *x_k_*. An approximation to the modulus of continuity is

$$\mu [f^{\prime \prime }(x);|\Delta_{k}|]≐\max\frac{\left|\frac{f[x_{k-1},x_{k}]}{h}-\frac{f[x_{k+1},x_{k}]}{h}\right|}{2h},$$ (6.2)

where f[] denotes the first order divided difference of *f*.

For a 7 mm air line with a mesh size (Δ*_k_*) equal to 1 mm, a value of *μ*, from observations of conductor radii measurements as functions of *z* indicate *μ*=0.1 is reasonable, and in the angular direction *μ* < 0.01.^7^ Therefore, the total error from spline interpolation is (considering the errors as additive)

$${\text{Error}}_{\text{total}}={\text{Error}}_{z}+{\text{Error}}_{\theta }<2.8{\times 10}^{-7}m.$$ (6.3)

The error source (c) from conformal mapping an elliptical measurement contour of eccentricity equal to 38.1×10^−6^ m (150 *μ*. in) is illustrated below.

First term of outer conductor equivalent (mapped radius) 3.4989 mm.

Second term 2.32×10^−12^ mm.

Third term −3.35×10^−12^ mm.

Keeping in mind that ellipses are symmetrical with respect to the origin reveals that even terms are effectively zero (on the order of 10^−12^ in view of machine precision). Since the convergence above is very strong, the fifth term is likely on the order of 10^−9^ mm. The error from source (d) depends on the number of expansion terms representing the functions 1/*a*(*z*), 1/*b*(*z*), and ln{*b*(z)/*a*(z)}.

At this point accuracy considerations of the forward and backward wave eqs (3.7) and (3.10) in correspondence to the measurement system are in order. For instance, to examine $A_{00}^{+}$ consider the measurements of *a*(*z*) and *b*(*z*) at *z*=0 and *z=z*_1_. From eqs (3.7) and (3.10) we have the total differentials

$$\Delta A_{00}^{+}(0;z_{1})≐A_{0}\left\{W(a_{0},b_{0},Z_{s})-\left[\frac{Z_{s}}{2k_{0}}U_{2,1}(a_{0},b_{0})-\frac{Z_{s}}{2k_{0}}U_{1,2}(a_{0},b_{0})\left[\frac{1}{a_{0}}+\frac{1}{b_{0}}\right]\right]\right\}e^{-W(a_{0},b_{0}Z_{s})z_{1}}\Delta r_{0}$$ (6.4)

and

$$\begin{array}{l} \Delta A_{00}^{-}(0;z_{1})=-A_{0}\left\{\frac{Z_{s}}{2k_{0}}U_{1,1}(a_{0},b_{0})+\frac{1}{\ln\frac{b_{0}}{a_{0}}}\left[\frac{b_{1}}{b_{0}}-\frac{a_{1}}{a_{0}}\right]\right\}\\ \cdot e^{-2W(a_{0},b_{0},z_{s})z_{1}}\Delta z_{1}+A_{0}\frac{Z_{s}}{2k_{0}}\{U_{1,1}(a_{0},b_{0})\left[\frac{b_{1}}{b_{0}}-\frac{a_{1}}{a_{0}}\right]\\ +\frac{1}{\ln\left[\frac{b_{0}}{a_{0}}\right]}\left[\frac{a_{1}}{a_{0}^{2}}-\frac{b_{1}}{b_{0}^{2}}\right]\}\cdot \frac{e^{-2W(a_{0},b_{0},Z_{0})z_{1}}-1}{2W(a_{0},b_{0},z_{s})}\Delta r_{0}\\ +A_{0}\frac{Z_{s}}{2k_{0}}\{U_{1,1}(a_{0},b_{0})\\ +\frac{1}{\ln\left[\frac{b_{0}}{a_{0}}\right]}\left[\frac{b_{1}}{b_{0}}-\frac{a_{1}}{a_{0}}\right]\}\{U_{2,1}(a_{0},b_{0})\\ -U_{1,2}(a_{0},b_{0})\left[\frac{1}{a_{0}}+\frac{1}{b_{0}}\right]\}\{\frac{1}{W^{2}(a_{0},b_{0},Z_{s})}\\ -\left[\frac{z_{1}}{W(a_{0},b_{0},Z_{s})}+\frac{1}{W^{2}(a_{0},b_{0},Z_{s})}e^{-W(a_{0},b_{0},Z_{s})z_{1}}\right]\}\Delta r_{0} \end{array}$$ (6.5)

where Δ*r*_0_ is the measurement precision common to *a*_0_ and *b*_0_. In the expressions above

$$U_{\ell ,k}(a_{0},b_{0})=\frac{1}{a_{0}^{\ell }}+\frac{1}{b_{0}^{\ell }}\frac{\;}{\ln^{k}\left[\frac{b_{0}}{a_{0}}\right]}$$ (6.6)

and

$$W(a_{0},b_{0},Z_{s})=\frac{Z_{z}}{2k_{0}}U_{1,1}(a_{0},b_{0})+\left\{\frac{b_{1}}{b_{0}}-\frac{a_{1}}{a_{0}}\right\}$$ (6.7)

where *a*_1_ and *b*_1_ are the coefficients from differentiating the cubic spline representations of *a*(*z*) and *b*(*z*), respectively.

In computing $\Delta A_{00}^{+}$ and $\Delta A_{00}^{-}$ for a 7 mm air line let the measurement precision be Δ*z*=Δ*r*_0_=2.8×10^−7^ m for a frequency range of 1–18 GHz. Using the measurements of *a*(z) and *b*(*z*) in figure 3, we select the maximum divided difference magnitudes for *a*_1_ and *b*_1_ to obtain the results

$$\begin{array}{l} \left|\Delta A_{00}^{+}(0;z_{1})\right|≐3.6\times 10^{-5}\text{at}\;1\;\text{GHz},\\ \;≐1.1\times 10^{-4}\text{at}\;18\;\text{GHz}, \end{array}$$ (6.8)

$$\begin{array}{l} \left|\Delta A_{00}^{-}(0;z_{1})\right|≐4\times 10^{-8}\text{at}\;1\;\text{GHz},\\ \;≐1.5\times 10^{-7}\text{at}\;18\;\text{GHz}. \end{array}$$ (6.9)

To examine the total uncertainty in $A_{00}^{\pm }$ over *N* measurements of *a*(*z_k_*) and *b*(*z_k_*) for *k* = 1,⋯,*N* let $\Delta A_{00}^{\pm }$ represented as

$$\Delta A_{00}^{\pm }(0;z_{N})≐\sum_{n=1}^{N}f\{a(z_{k}),b(Z_{n})\}\Delta A_{00}^{\pm }(z_{n-1},z_{n}),$$ (6.10)

where *f_n_* is on the order of $A_{00}^{\pm }(z_{n-1};z_{n})$. Since the computation of $\Delta A_{00}^{\pm }(z_{n-1};z_{n})$ proceeds in the same way as $\Delta A_{00}^{\pm }(0;z_{1})$, in eq (6.5), the total imcertainty over all measurement positions is found when the individual uncertainties in *z* and *r*_0_ at each measurement position are known.

### 7.2 Efficiency Improvements in Cubic Spline Approximation

While fitting the surfaces of coaxial air line geometries with products of cubic splines over the variables *θ* and z successfully meets error bounds consistent with measurement precision, significant reductions in the number of measurements yields equivalent error bounds with Gordon’s successive decomposition spline [18]. The number of measurements required for successive decomposition splines in comparison to usual spline products is generally less than 50 percent.

## 8. Summary

Generalized telegraphist equations for the coaxial air line have been derived under two assumptions: (a) skin effect losses are present, and (b) conductor surface variations occur in the axial and transverse coordinates. Product cubic spline expressions to accurately fit conductor surface measurements were employed to arrive at pointwise scattering parameter expressions. Error bounds from eqs (6.8) and (6.9) reveal at least four significant figures can be obtained to characterize the scattering parameters *S*_21_ and *S*_11_ in correspondence to a conductor surface measurement resolution of 2.8×10^−7^ m over frequencies appropriate to the principal mode for 7 mm air lines.

## Figures and Tables

**Figure 6:**
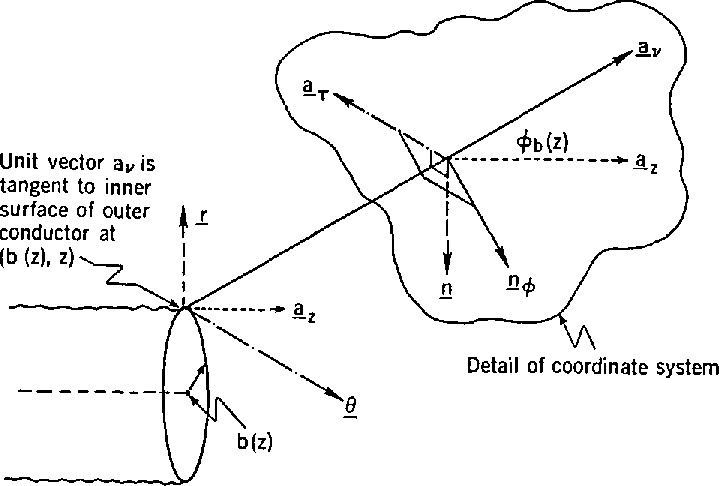
Outer conductor coordinate systems for determining boundary conditions.

## Footnotes

1 Since *S*(*z*) is differentiable and the field components are continuous, interchange of differentiation and integration is justified.

2 Higher order mode influence on the TEM mode will be reported in a later issue of this journal.

3 In the lossless air line *T*_00_(z)=0 in view of the eqs (2.5) and (2.6).

4 A cursory inspection of calculated splines *C_k_* over the entire length of the line reveals that ${\hat{C}}_{0k}$ is not necessarily equal to the radial measurement at *z_k_*_−1_.

5 For additional computational results see Holt [17].

6 See Davis [16] for a suitable definition.

7 Since mechanical measurements for roundness are not available, an ellipse of 38.1 × 10^−6^ m was selected for determining the modulus of continuity.

## Appendix A. Inner and Outer Conductor Boundary Conditions

In reference to figure 6 the boundary condition is

$$E_{v}=z_{s}J_{v}$$ (A.1)

where *E_v_* is the electric field component, *J_v_* is the current density component in the coordinate *v*, and *Z_s_* is the radial impedance at *r* = *b*(*z*).

Also from figures 1 and 6

$$\begin{array}{l} a_{v}=\left|a_{v}\right|\cos\{\phi_{b}(z)\}a_{z}+\left|a_{v}\right|\sin\{\phi_{b}(z)\}(-n),\\ \;a_{v}=\cos\{\phi_{b}(z)\}a_{z}-\sin\{\phi_{b}(z)\}n, \end{array}$$ (A.2)

where **n** is the unit outward normal vector. From the left side of eqs (A.1) and (A.2)

$$\begin{array}{l} E_{v}a_{v}=\left|E_{v}\right|\cos\{\phi_{b}(z)\}a_{z}-\left|E_{v}\right|\sin\{\phi_{b}(z)\}n,\\ \;E_{v}a_{v}=E_{z}a_{z}+E_{r}a_{r}. \end{array}$$

Proceeding with the right side of eq (A.1), yields

$$J_{v}a_{v}=J_{z}a_{z}-J_{n}n=a_{z}\cdot n\times H-J_{n}n.$$ (A.4)

To find an expression for *J_n_* note that

$$J_{n}=n\cdot J_{v}=n\cdot n_{\phi }\times H,$$ (A.5)

$$\begin{array}{l} J_{n}=n\cdot n_{\phi }\times \{H_{\tau }a_{\tau }+H_{n}\;n+H_{z}a_{z}\},\\ J_{n}=n\cdot n_{\phi }\times \{H_{\tau }a_{\tau }+H_{n}\;\{-\sin\{\phi_{b}(z)\}a_{v}\\ \;+\cos\{\phi_{b}(z)\}n_{\phi }\}.\\ \;+H_{z}\{\cos\{\phi_{b}(z)\}a_{v}+\sin\{\phi_{b}(z)\}n_{\phi }\},\\ J_{n}=-\sin\{\phi_{b}(z)\}H_{\tau }. \end{array}$$ (A.6)

Consolidating eqs (A.3), (A.4), and (A.6) yields

$$E_{z}=-Z_{s}\left\{\frac{1+\sin\{\phi_{b}(z)\}}{1+\tan\{\phi_{b}(z)\}}\right\}H_{\theta }$$ (A.7)

where the relation *E_z_* tan*ϕ*(*z*) = *E_r_*, has been employed.

The inner conductor boundary condition relation is similarly derived as follows: We have *E_v_=Z_s_ J_v_* and the left hand side is

$$E_{v}=E_{z}+E_{r}.$$ (A.8)

The right side becomes

$$a_{v}J_{v}=a_{z}J_{z}+a_{r}J_{r}.$$ (A.9)

Now proceeding as before yields

$$J_{r}=a_{r}\cdot J_{v}=a_{r}\cdot n_{\phi }\times H,$$ (A.10)

$$\begin{array}{l} J_{r}=a_{r}\cdot H_{\theta }a_{v},\\ J_{r}=\sin\{\phi_{a}(z)\}H_{\theta }. \end{array}$$ (A.11)

Substituting *E_r_* = tan{*ϕ_a_*(*z*)} *E_r_* into eq (A.8) and using eq (A.11) in (A.9) yields

$$E_{z}=Z_{s}\frac{1+\sin\{\phi_{a}(z)\}}{1+\tan\{\phi_{a}(z)\}}H_{\theta }.$$ (A.12)

## Appendix B. Transformation of Basis Functions for the Field Components

The transverse fields *E_r_* and **H***_θ_* are represented as

$$\begin{array}{l} E_{r}(r,\theta ,z)=\sum_{n,p}\frac{\partial }{\partial r}\Phi_{np}(r,z)\{{\hat{V}}_{np}^{\left(1\right)}(z)\cos(n\;\theta )\\ \;+{\hat{V}}_{np}^{\left(2\right)}(z)\sin(n\;\theta )\} \end{array}$$ (B.1)

$$\begin{array}{l} H_{\theta }(r,\theta ,z)=\sum_{n,p}\frac{\partial }{\partial r}\Phi_{np}(r,z)\{{\hat{I}}_{np}^{\left(1\right)}(z)\cos(n\;\theta )\\ \;+{\hat{I}}_{np}^{\left(2\right)}(z)\sin(n\;\theta )\} \end{array}$$ (B.2)

where Φ*_np_* denotes the radial component of the normalized potential function at a point, *z*. The symbol ˆ over *V_np_* (or *I_np_*) signifies an amplitude corresponding to the nonorthogonal function $\frac{\partial }{\partial r}\Phi_{np}$ and the superscripts (1) [and (2)] correspond to even and odd modes. We assume that *E_r_* and *H_θ_* have first and second derivatives in *r* and *θ* which are continuous and bounded. Hence, the Fourier series eqs (B.1) and (B.2) are absolutely and uniformly convergent; that is the series can be rearranged.

Suppose the *k*th even mode of eqs (B.1) and (B.2) is considered and let the Gram-Schmidt orthogonalization be represented as

$$\frac{\partial }{\partial r}\Phi_{np}(r,z)=\sum_{\ell =0}^{p}a_{k,p-1,\ell }(z)f_{k\ell }(r,z)/N_{k\ell }(z)$$ (B.3)

where *f_kℓ_* (*r,z*)/*N_kℓ_*(*z*) is the *k ℓ* th orthogonal basis function. Now substituting eq (B.3), multiplying eq (B.1) by cos(k*θ*), and integrating over *θ* yields

$$\begin{array}{l} \int_{0}^{2\pi }E_{r}(r,\theta ,z)\cos(k\theta )d\theta \\ =\{{\hat{V}}_{k0}(z)a_{k,0,0}(z)f_{k0}(r,z)/N_{k0}(z)\}\\ +{\hat{V}}_{k1}(z)\{a_{k,1,0}(z)\frac{f_{k0}(r,z)}{N_{k0}(z)}+a_{k,1,1}(z)\frac{f_{k1}(r,z)}{N_{k1}(z)}\}+\cdots \\ +{\hat{V}}_{km}(z)\{a_{k,m,0}(z)\frac{f_{k0}(r,z)}{N_{k0}(z)}\\ +\cdot \cdot +a_{k,m,m}(z)\frac{f_{km}(r,z)}{N_{km}(z)}\}+\cdots \}v(k). \end{array}$$ (B.4)

By induction note that the basis function *f_k_*_0_(*r,z*)/*N_k_*_0_(*z*), occurs in every term above. Hence, we set

$$V_{k,0}^{\left(1\right)}(z)=\sum_{\ell =0}^{\infty }a_{k,\ell ,0}(z){\hat{V}}_{k\ell }(z).$$

Proceeding to the *m* th term yields the expression

$$V_{km}^{\left(1\right)}(z)=\sum_{\ell =m}^{\infty }a_{k,\ell ,m}(z){\hat{V}}_{k\ell }(z)$$ (B.5)

for the coefficient of the basis function *f_km_*(*r,z*)/*N_km_*(*z*). Now eq (B.4) from eq (B.5) becomes

$$\begin{array}{l} \int_{0}^{2\pi }E_{r}(r,\theta ,z)\cos k\theta d\theta \\ \;=v(k)\sum_{p=0}^{\infty }V_{kp}^{\left(1\right)}(z)f_{kp}(r,z)/N_{kp}(z) \end{array}$$ (B.6)

for the *kth* even component of *E_r_.* The odd components of *E_r_* are obtained similarly. Hence, summing all components of *E_r_* obtains the desired result

$$\begin{array}{l} E_{r}(r,\theta ,z)=v(n)\sum_{n,p}\frac{f_{np}(r,z)}{N_{np}(z)}\{V_{np}^{\left(1\right)}(z)\cos(n\;\theta )\\ +V_{np}^{\left(2\right)}(z)\sin(n\;\theta )\} \end{array}$$ (B.7)

where

$$\begin{array}{l} v(n)=2\pi \;\text{if}\;n=0\\ \;=\pi \;\text{if}\;n\neq 0 \end{array}$$

The expansion for *H_θ_* is obtained in the same way, i.e.,

$$\begin{array}{l} H_{\theta }(r,\theta ,z)=v(n)\sum_{n,p}\frac{f_{np}(r,z)}{N_{np}(z)}\{I_{np}^{\left(1\right)}(z)\cos(n\;\theta )\\ \;+I_{np}^{\left(2\right)}(z)\sin(n\;\theta )\}. \end{array}$$ (B.8)

## Appendix C

Beginning with the spline expressions eq (3.1), consider the reciprocal

$$\frac{1}{b(z)}=\frac{1}{b_{0}}\frac{1}{1+\frac{b_{1}}{b_{0}}z+\cdot \cdot +\frac{b_{3}}{b_{0}}z^{3}}.$$ (C.1)

We define the coefficients

$$B_{1}^{\left(0\right)}=b_{1}/b_{0},\ldots ,B_{3}^{\left(0\right)}=b_{3}/b_{0}.$$ (C.2)

Since the geometric series for small *z*,

$$\left|\frac{b_{1}}{b_{0}}z+\cdots +\frac{b_{3}}{b_{0}}z^{3}\right|⩽\alpha_{0}<<1$$

converges uniformly and absolutely, eq (C.1) can be rearranged in powers of *z*. We have

$$\frac{1}{b(z)}=\frac{1}{b_{0}}\frac{1}{1+B_{1}^{\left(0\right)}z+\cdot \cdot +B_{3}^{\left(0\right)}z^{3}}.$$

Using the geometric expansion yields

$$\frac{1}{b(z)}=\frac{1}{b_{0}}\{1-B_{1}^{\left(0\right)}z-\cdot \cdot -B_{3}^{\left(0\right)}z^{3}\}+\{{\left(B_{1}^{\left(0\right)}\right)}^{2}z^{2}+2B_{1}^{\left(0\right)}B_{2}^{\left(0\right)}z^{3}+\cdots \}-{\left[B_{2}^{\left(0\right)}\right]}^{3}z^{3}-\cdots \},$$ (C.3a)

and rearranging the above series into successive powers of *z* produces

$$\frac{1}{b(z)}=\frac{1}{b_{0}}+D_{b1}^{\left(0\right)}z+D_{b2}^{\left(0\right)}z^{2}+D_{b3}^{\left(0\right)}z^{3}\cdots$$ (C.3b)

where

$$\begin{array}{l} D_{b1}^{\left(0\right)}=-B_{1}^{\left(0\right)},D_{b2}^{\left(0\right)}=-B_{2}^{\left(0\right)}+{\left[B_{1}^{\left(0\right)}\right]}^{2}\\ D_{b3}^{\left(0\right)}=-B_{3}^{\left(0\right)}+2B_{1}^{\left(0\right)}B_{2}^{\left(0\right)}-{\left\{B_{1}^{\left(0\right)}\right\}}^{2}. \end{array}$$ (C.4)

To obtain an expression for ln{*b*(*z*)/*a*(*z*)} consider the expression

$$\ln\left[\frac{b(z)}{a(z)}\right]=\ln\frac{b_{0}}{a_{0}}+\ln\{1+B_{1}^{\left(0\right)}z+\cdot \cdot +B_{3}^{\left(0\right)}z^{3}\}-\ln\{1+A_{1}^{\left(0\right)}z+\cdot \cdot +A_{3}^{\left(0\right)}z^{3}\}$$ (C.5)

Taking the reciprocal of eq (C.5) and using the geometric expansion yields

$$\begin{array}{l} \ln{\left[\frac{b(z)}{a(z)}\right]}^{-1}={\left\{\ln\frac{b_{0}}{a_{0}}\right\}}^{-1}\{1-{\left\{\ln\frac{b_{0}}{a_{0}}\right\}}^{-1}\{\ln[1+B_{1}^{\left(0\right)}z\\ +\cdot \cdot ]+\ln[1+A_{1}^{\left(0\right)}z+\cdot \cdot ]\}\\ +{\left\{\ln\frac{b_{0}}{a_{0}}\right\}}^{-2}\{\ln^{2}(1+B_{1}^{\left(0\right)}z+\cdot \cdot )\\ -2\ln[1+B_{1}^{\left(0\right)}z+\cdot \cdot ]\ln[1+A_{1}^{\left(0\right)}z+\cdot \cdot ]\\ +\ln^{2}[1+A_{1}^{\left(0\right)}z+\cdot \cdot ]\}. \end{array}$$ (C.6)

From the absolutely and uniformly convergent expansion

$$\ln\{1+\omega \}=\omega -\frac{\omega^{2}}{2}+\frac{\omega^{3}}{3}+\cdots$$

for |ω|⩽*α*_0_< <1

the expansion eq (C.6) is rearranged in powers of *z* to obtain

$${\left\{\ln\frac{b(z)}{a(z)}\right\}}^{-1}=\sum_{n=0}^{\infty }H_{n}^{\left(1\right)}z^{n}$$ (C.7)

where the first four coefficients are defined as

$$\begin{array}{l} H_{0}^{\left(1\right)}={\left\{\ln\frac{b_{0}}{a_{0}}\right\}}^{-1}\\ H_{1}^{\left(1\right)}=-{\left\{\ln\frac{b_{0}}{a_{0}}\right\}}^{-2}\{G_{b0}^{\left(1\right)}-G_{a0}^{\left(1\right)}\}\\ H_{2}^{\left(1\right)}=-{\left\{\ln\frac{b_{0}}{a_{0}}\right\}}^{-2}\{G_{b1}^{\left(1\right)}-G_{a1}^{\left(1\right)}\}\\ +{\left\{\ln\frac{b_{0}}{a_{0}}\right\}}^{-3}\{G_{b0}^{\left(2\right)}-2G_{b0}^{\left(1\right)}G_{a0}^{\left(1\right)}+G_{a0}^{\left(2\right)}\}\\ H_{3}^{\left(1\right)}=-{\left\{\ln\frac{b_{0}}{a_{0}}\right\}}^{-2}\{G_{b2}^{\left(1\right)}-G_{a2}^{\left(1\right)}\}\\ +{\left\{\ln\frac{b_{0}}{a_{0}}\right\}}^{-3}\cdot \{G_{b1}^{\left(2\right)}-2G_{b1}^{\left(2\right)}G_{a0}^{\left(1\right)}-2G_{b0}^{\left(1\right)}G_{a1}^{\left(1\right)}+G_{a1}^{\left(2\right)}\}\\ -{\left\{\ln\frac{b_{0}}{a_{0}}\right\}}^{-4}\{G_{b0}^{\left(3\right)}-3G_{b0}^{\left(2\right)}G_{a0}^{\left(1\right)}+3G_{b0}^{\left(1\right)}G_{a0}^{\left(2\right)}+G_{a0}^{\left(3\right)}\} \end{array}$$ (C.8)

and the G coefficients for the outer conductor cue

$$\begin{array}{l} G_{b0}^{\left(k\right)}={\left\{B_{1}^{\left(0\right)}\right\}}^{k};k=1,2,3.\\ G_{b1}^{\left(1\right)}=B_{2}^{\left(0\right)}-\frac{{\left\{B_{1}^{\left(0\right)}\right\}}^{2}}{2},\\ G_{b1}^{\left(2\right)}=2G_{0}^{\left(1\right)}G_{1}^{\left(1\right)}=2B_{1}^{\left(0\right)}\left\{B_{2}^{\left(0\right)}-\frac{{\left[B_{1}^{\left(0\right)}\right]}^{2}}{2}\right\},\\ G_{b2}^{\left(1\right)}=B_{3}^{\left(0\right)}-B_{1}^{\left(0\right)}B_{2}^{\left(0\right)}+\frac{{\left[B_{1}^{\left(0\right)}\right]}^{3}}{3}. \end{array}$$ (C.9)

The *G* coefficients for the inner conductor are computed by replacing *B_k_*(0) with *A_k_*(0). To obtain the expansion for *κ* in eq (1.15) an expression for *K*[*ϕ*(z)] needs to be found. From eqs (3.1) and (1.1)

$$\begin{array}{l} K[\phi_{b}(z)]=\frac{1+\sin[\phi_{b}(z)]}{1+\tan[\phi_{b}(z)]},\\ K[\phi_{b}(z)]=\frac{1+b^{\prime }(z)/{\left\{1+b^{\prime }{\left(z\right)}^{2}\right\}}^{2}}{1+b^{\prime }(z)}. \end{array}$$ (C.10)

Using the binomial and geometric expansions, in the last expression, yields the following expression in terms of spline coefficients

$$K[\phi_{b}(z)]=1-\frac{3}{2}b_{1}^{2}-6b_{1}b_{2}z.$$

We now have from eqs (C.3), (C.7), and (CIO) the expression

$$\begin{array}{l} \frac{K\{\phi_{b}(z)\}}{b(z)\ln\frac{b(z)}{a(z)}}=\{1-\frac{3}{2}b_{1}^{2}\\ -6b_{1}b_{2}z\}\sum_{0}^{3}D_{n}^{\left(0\right)}z^{n}\sum_{0}^{3}H_{m}^{\left(1\right)}z^{m}\dot{=}\sum_{0}^{3}C_{bk}^{\left(1\right)}z^{k} \end{array}$$ (C.11)

where $C_{k}^{\left(1\right)}$ is the convolution of coefficients, i.e.,

$$\begin{array}{l} C_{b0}^{\left(1\right)}=\left(1-\frac{3}{2}b_{1}^{2}\right)D_{0}^{\left(0\right)}H_{0}^{\left(1\right)}\\ C_{b1}^{\left(1\right)}=\left(1-\frac{3}{2}b_{1}^{2}\right)\{D_{0}^{\left(0\right)}H_{1}^{\left(1\right)}+H_{0}^{\left(1\right)}D_{1}^{\left(0\right)}\}\\ -6b_{1}b_{2}D_{0}^{\left(0\right)}H_{0}^{\left(1\right)}\\ C_{b2}^{\left(1\right)}=\left(1-\frac{3}{2}b_{1}^{2}\right)\sum_{k=0}^{2}D_{2-k}^{\left(0\right)}H_{k}^{\left(1\right)}-6b_{1}b_{2}\sum_{k=0}^{1}D_{1-k}^{\left(0\right)}H_{k}^{\left(1\right)}\\ C_{b3}^{\left(1\right)}=\left(1-\frac{3}{2}b_{1}^{2}\right)\sum_{k=0}^{2}D_{3-k}^{\left(0\right)}H_{k}^{\left(1\right)}-6b_{1}b_{2}\sum_{k=0}^{1}D_{2-k}^{\left(0\right)}H_{k}^{\left(1\right)}. \end{array}$$ (C.12)

Since the inner conductor expansion proceeds in the same way, the total expression is

$$\kappa (z)\dot{=}\sum_{k=0}^{3}C_{k}^{\left(2\right)}z^{k}$$ (C.13)

where

$$C_{k}^{\left(2\right)}=C_{ak}^{\left(1\right)}+C_{bk}^{\left(1\right)}.$$ (C.14)

The transfer coefficient eq (2.4) has the expansion

$$\begin{array}{l} T_{00}(z)=[b_{1}+2b_{2}z+3b_{3}z^{2}][\sum_{k=0}^{3}D_{bk}^{\left(0\right)}z^{k}][\sum_{k=0}^{3}H_{bk}^{\left(1\right)}z^{k}]\\ -[a_{1}+2a_{2}z+3a_{3}z^{2}]\sum_{k=0}^{3}D_{ak}^{\left(0\right)}z^{k}\sum_{k=0}^{3}H_{ak}^{\left(1\right)}z^{k},\\ T_{00}(z)=\sum_{k=0}^{3}C_{k}^{\left(3\right)}z^{k}. \end{array}$$ (C.16)

where

$$\begin{array}{l} C_{n}^{\left(3\right)}\equiv \sum_{m=\mu (n)}^{n}\sum_{k=0}^{m}\{(n╪1=m)b_{n+1-m}D_{bm-k}^{\left(0\right)}H_{k}^{\left(1\right)}\\ -(n+1-m)a_{n+1-m}D_{a,m-k}^{\left(0\right)}H_{k}^{\left(1\right)}\} \end{array}$$

and

$$\begin{array}{l} \mu (n)=0\;\text{for}\;n<3,\\ \;=1\;\text{for}\;n=3. \end{array}$$ (C.17)

From eqs (2.3), (C.14), and (C.17) set

$$\delta_{0}^{\left(1\right)}=j\beta +\frac{Z_{s}}{2k_{0}}C_{0}^{\left(2\right)}$$ (C.18)

$$\delta_{k}^{\left(1\right)}=\frac{Z_{s}}{2k_{0}}C_{k}^{\left(2\right)};k⩾1$$ (C.19)

$$\delta_{k}^{\left(2\right)}=\frac{Z_{s}}{2k_{0}}C_{k}^{\left(2\right)}+\frac{C_{k}^{\left(3\right)}}{2};k⩾0.$$ (C.20)

The coefficients $v_{k,m-1}^{\left(2\right)}$ in eq (3.14) are defined by selecting the coefficients of *η^k^* and convoluting the series product. Let

$$\begin{array}{l} v_{0,m-1}^{\left(1\right)}=1\\ v_{1,m-1}^{\left(1\right)}=0\\ v_{2,m-1}^{\left(1\right)}=-\delta_{1,m-1}^{\left(1\right)}\\ v_{3,m-1}^{\left(1\right)}=-\frac{2}{3}\delta_{2,m-1}^{\left(1\right)}\\ v_{4,m-1}^{\left(1\right)}=-\frac{1}{2}\delta_{3,m-1}^{\left(1\right)}+\frac{1}{2}{\left\{\delta_{1,m-1}^{\left(1\right)}\right\}}^{2}\\ v_{5,m-1}^{\left(1\right)}=\frac{2}{3}\delta_{1,m-1}^{\left(1\right)}\delta_{2,m-1}\\ v_{6,m-1}^{\left(1\right)}=2\left\{\frac{\left\{\delta_{2,m-1}^{\left(1\right)}\right\}}{3^{2}}+\frac{1}{4}\delta_{1,m-1}^{\left(1\right)}\delta_{3,m-1}^{\left(1\right)}\right\}\\ v_{7,m-1}^{\left(1\right)}=\frac{1}{3}\delta_{2,m-1}^{\left(1\right)}\delta_{3,m-1}^{\left(1\right)}\\ v_{8,m-1}^{\left(1\right)}=\frac{1}{8}{\left\{\delta_{3,m-1}^{\left(1\right)}\right\}}^{2}-\frac{1}{6}{\left\{\delta_{1,m-1}^{\left(1\right)}\right\}}^{3}. \end{array}$$ (C.21)

Proceeding to $v_{k,m-1}^{\left(2\right)}$

$$v_{k,m-1}^{\left(2\right)}=\sum_{\ell =0}^{\mu_{1}(k)}\delta_{k-\ell ,m-1}^{\left(2\right)}v_{\ell ,m-1}^{\left(1\right)}$$ (C.22)

where

$$\begin{array}{l} \mu_{1}(k)=k\;\text{if}\;0⩽k⩽3\\ \;=3\;\text{if}\;3<k⩽8 \end{array}$$ (C.23)

and

$$v_{k,m-1}^{\left(2\right)}=\sum_{\ell =k-8}^{3}\delta_{k-\ell ,m-1}^{\left(2\right)}v_{\ell ,m-1}^{\left(1\right)}\;\text{if}\;8<k⩽11.$$ (C.24)

## Appendix D

Expression of the contour length *ℓ*_0_ in terms of the spline coefficients is initiated from the definition

$$\ell_{0}=\int_{0}^{2\pi }\left\{\zeta (\theta )+\zeta^{-2}(\theta ){\left[\frac{\partial \zeta }{\partial \theta }\right]}^{2}-..\right\}d\theta .$$ (D.1)

Using the spline coefficients for the interval [θ*_n_*, θ*_n+_*_1_] yields the representation

$$P_{n}(\eta )=\sum_{k=0}^{3}P_{1,k,n}^{\left(1\right)}\eta^{k},0⩽\eta ⩽\theta_{n+1}$$ (D.2)

at *n*=0, $P_{n}\left(0\right)=P_{1,0,n}^{\left(1\right)}=$the measurement of *ζ* at *θ = θ_n_.* Then eq (D.1), in view of eq (D.2), yields

$$\begin{array}{l} 1_{0}=\sum_{n=0}^{N-1}\int_{0}^{\theta_{n+1}-\theta_{n}}\{\sum_{k=0}^{3}P_{1,k,n}^{\left(1\right)}\theta^{k}\\ +\frac{1}{2}{\left[\sum_{k=0}^{3}(k+1)P_{1,k+1,n}^{\left(1\right)}\theta^{k}\right]}^{2}\frac{1}{\sum_{k=0}^{3}P_{1,k,n}^{\left(1\right)}\theta^{k}}\}d\theta . \end{array}$$ (D.3)

The denominator can be expanded into a geometric series such that the second term above yields

$$\begin{array}{l} \sum_{k=0}^{7}P_{2,k,n}^{\left(1\right)}\theta^{k}\dot{=}{\left\{\sum_{k=0}^{3}(k+1)P_{1,k+1,n}^{\left(1\right)}\theta^{k}\right\}}^{2}\left\{\frac{1}{P_{1,0,n}^{\left(1\right)}}\right\}\\ \cdot \left\{1-\frac{p_{1,1,n}^{\left(1\right)}}{p_{1,0,n}^{\left(1\right)}}\theta -\cdot \cdot -\frac{p_{3,1,n}^{\left(1\right)}}{p_{1,0,n}^{\left(1\right)}}+\cdot \cdot \right\} \end{array}$$ (D.4)

and eq (D.3) on substituting eq (D.4) yields the form eq (4.13). For other values of *p* and *q* we have

$$\begin{array}{l} h_{pq}=\frac{1}{\ell_{0}}\sum_{n=0}^{N-1}e^{i(p-q)\theta_{n}}\int_{0}^{\theta_{n+1}-\theta_{n}}\{\sum_{k=0}^{3(p+q+1)}P_{1,k,n}^{\left(p+q+1\right)}\theta^{k}\\ +1/2\left[\sum_{k=0}^{3(p+q+1)}P_{1,k,n}^{\left(p+q-1\right)}\theta^{k}\right]{\left[\sum_{k=0}^{2}(k+1)P_{1,k+1,n}^{\left(1\right)}\theta^{k}\right]}^{2}\}\\ \cdot e^{i(p-q)\theta }d\theta \end{array}$$ (D.5)

where

$$\sum_{k=0}^{3(p+q)}P_{1,k,n}^{\left(p+q\right)}\theta^{k}=\left\{\sum_{k=0}^{3}P_{1,k,n}^{\left(1\right)}\theta^{k}\right\}.$$ (D.6)

Let $P_{2,k,n}^{\left(p+q-1\right)}$ be the convolution of the product $P_{2,k,n}^{\left(p+q-1\right)}$ and ${\left\{(k+1)P_{1,k+1,n}^{\left(1\right)}\right\}}^{2}$ above. Then eq (D.5) yields

$$\begin{array}{l} h_{pq}=\frac{1}{\ell_{0}}\sum_{n=0}^{N-1}e^{i(p-q)\theta_{n}}\sum_{k=0}^{3(p+q+1)}P_{1,k,n}^{\left(p+q+1\right)}\left\{S_{k}[(\theta_{n+1}-\theta_{n})\right\}\\ \cdot (p-q)j]-S_{k}(0)\}+\frac{1}{2}\sum_{k=0}^{3(p+q+1)}\{e^{i(p-q)}\theta_{n}\}P_{2,k,n}^{\left(p+q+1\right)}\\ \cdot \{S_{k}[(\theta_{n+1}-\theta_{n})(p-q)j]-S_{k}(0)\} \end{array}$$ (D.7)

where *S_k_* is defined in eq (3.11).
