# Erratum: Scattering Parameters Representing Imperfections in Precision Coaxial Air Lines

**DOI:** 10.6028/jres.094.030

**Published:** 1989

**Authors:** Donald R. Holt

**Affiliations:** National Institute of Standards and Technology, Boulder, CO 80303

[J. Res. Natl. Inst. Stand. Technol., Volume 94, Number 2, March-April 1989, p. 117]

The following errors were found in this paper: On page 121, eq (2.7b) should read:

$$\frac{dA_{00}^{-}}{dz}-j\beta A_{00}^{-}=-\frac{1}{2}\frac{1}{\ln\frac{b\left(z\right)}{a\left(z\right)}}\left\{\frac{b^{\prime }\left(z\right)}{b\left(z\right)}-\frac{a^{\prime }\left(z\right)}{a\left(z\right)}\right\}A_{00}^{+}.$$

On page 121, the equation immediately above eq (3.2c) should read as follows:

$$C_{k-1}\left(0\right)={\hat{C}}_{k-1}\left(z_{k-1}\right).$$

On page 121, the sentence immediately below eq (3.2c) should read:such that C_0_,*_k_*_−1_ represents the measurement of *a*(*z*) or *b*(*z*) at *z* = *z_k_*_−1_.

On page 122, eq (3.8) should read:

$$A_{00}^{-}\left(0;z\right)=A_{00}^{-}\left(0;z_{1}\right)-A_{00}^{+}\left(0;z_{1}\right)\{e^{-j\beta z_{1}\int^{z-z_{1}}}\ldots .$$

On page 129, the sentence immediately below eq (A. 11) should read:Substituting E*_r_* = tan{ *ϕ_a_*(*z*)} *E_z_*….

On page 133, eq (D.3) should read:

$$\ell_{0}=\sum_{n=0}^{N-1}\ldots .$$

